# *PyWRKY26* and *PybHLH3* cotargeted the *PyMYB114* promoter to regulate anthocyanin biosynthesis and transport in red-skinned pears

**DOI:** 10.1038/s41438-020-0254-z

**Published:** 2020-03-15

**Authors:** Chuang Li, Jun Wu, Kang-Di Hu, Shu-Wei Wei, Hong-Ye Sun, Lan-Ying Hu, Zhuo Han, Gai-Fang Yao, Hua Zhang

**Affiliations:** 1 School of Food and Biological Engineering, Hefei University of Technology, 230009 Hefei, China; 2 Centre of Pear Engineering Technology Research, State Key Laboratory of Crop Genetics and Germplasm Enhancement, Nanjing Agricultural University, 210095 Nanjing, China; 3 Shandong Institute of Pomology, 271000 Taian, China; 4 Anhui Province Key Laboratory of Functional Compound Seasoning, Anhui Qiangwang Seasoning Food Co., Ltd., 236500 Jieshou, China

**Keywords:** Transcriptional regulatory elements, Gene regulation

## Abstract

Red pear is favored because of its bright appearance and abundant anthocyanins. Anthocyanin biosynthesis is controlled by transcription factors (TFs) forming regulatory complexes. In red-skinned pears, the WRKY TFs have a significant relationship with anthocyanin biosynthesis, but the molecular mechanism of the WRKY TFs involved in regulating color formation in red-skinned pear is unclear. In this study, the TFs *PyWRKY31* and *PyWRKY26* were screened as candidate genes for controlling anthocyanin biosynthesis by transcriptome data and bioinformatics analysis. The effect of anthocyanin accumulations after cotransformation of *PyWRKY31* or *PyWRKY26* with its partners *PyMYB10*, *PyMYB114*, and *PybHLH3* was verified in tobacco leaves and strawberry receptacles by a transient expression system. RT-qPCR analysis and a dual-luciferase reporter system further confirmed that this cotransformation activated the expression of *PyDFR*, *PyANS*, and *PyUFGT* in anthocyanin biosynthesis and *PyGST* in anthocyanin transport instead of the *PyABC transporter* and *PyAVP*. Furthermore, the cotransformed *PyWRKY26* and *PybHLH3* could bind to the *PyMYB114* promoter, and *PyWRKY26* directly activated the transcription of *PyMYB114*. In addition, the TF PyWRKY26 could interact with PybHLH3, as confirmed by firefly luciferase complementation and yeast two-hybrid (Y2H) assays. These results showed that the interaction of PyWRKY26 and PybHLH3 could cotarget the *PyMYB114* promoter, which resulted in anthocyanin accumulation in red-skinned pear. This study further strengthened the understanding of the regulatory mechanism of anthocyanin accumulation and contributed to improving the appearance of red-skinned pears.

## Introduction

Pear (*Pyrus* L.) is one of the most common and popular fruits in the world, and red pears are favored by consumers for their beautiful appearance and abundant anthocyanins^1^. In plant tissues, anthocyanins are widely present; these molecules are important flavonoids with multiple physiological functions that aid pollination, seed dispersal, and resistance to adverse environmental conditions^2^. In addition, anthocyanins have significant antioxidant activity and potential benefits for human health, such as reducing the risk of cancer, inflammation, and coronary arteriosclerosis^3–5^.

Anthocyanin biosynthesis is coordinately regulated by structural genes and TFs in many species. The structural genes consist of seven enzyme genes, from phenylalanine ammonia-lyase (PAL) to UDP-glucose: flavonoid 3-*O*-glucosyltransferase (UFGT). In addition, synthetic anthocyanins are transported and stored in vacuoles. There are three mechanisms for anthocyanin transport: glutathione S-transferase (GST)-mediated transport, membrane transport, and vesicle trafficking^6,7^. GST can mobilize anthocyanins by acting as a carrier of these molecules, and anthocyanins are transported from the cytoplasm to the tonoplast through the ABC transmembrane transporter^8,9^. Many secondary transporters and channels, such as malate transporter and MATE-type transports and ABC transporters, were reported to exist on the tonoplast, and the activities of these transporters are directly or indirectly dependent on the proton gradient generated by the pyrophosphate-energized vacuolar membrane proton pump^9–11^.

By forming the MBW transcriptional complex, the MYB, bHLH, and WD40 proteins can regulate structural genes^12–14^. MYBs, as central factors regulating anthocyanin biosynthesis, have been widely researched. In horticultural crops, apple *MdMYB10*, *MdMYB1*, and *MdMYB110a*; strawberry *FaMYB10*; bayberry *MrMYB1*; and pear *PyMYB10*, and *PyMYB114* were reported to regulate anthocyanin biosynthesis in succession by forming the MBW transcriptional complex^15–18^. However, in different species, the regulatory mechanism of the MBW complex is different. In *Arabidopsis* and *Myrica rubra*, the three proteins in the MBW complex can interact with each other to form a transcriptional complex^19,20^. In apple, *MdMYB10* interacts with *MdbHLH3* and *MdbHLH33* to enhance the synthesis of anthocyanins^15^. In pears, *PyMYB114* or *PyMYB10* interacts with *PybHLH3* to significantly enhance anthocyanin biosynthesis^18^. Recently, overexpression of the *SlMYB75* gene was shown to promote anthocyanin biosynthesis in tomato^21^.

In addition to the MBW complex, other TFs, such as NACs, ERFs, HY5, BBX22, and WRKY, were found to be involved in regulating anthocyanin biosynthesis^22–24^. These TFs could regulate anthocyanin biosynthesis by indirectly or directly binding to the MBW complex. The WRKY TFs belong to one large gene family that regulates a series of physiological processes, including development and senescence, and resistance to adverse environments^24^. WRKY TFs were identified by one or two conserved WRKY domain(s) usually followed by a zinc-finger motif. The WRKY TF contains a specific nucleic acid sequence (C/T)TGAC(T/C) named the W-box, which regulates the defense response to various stresses by self-regulation, and it could recognize and bind to the W-box or other promoters of the WRKY TFs to generate biological effects by achieving crosstalk of different WRKY^25^. Compared with the MYB TFs, WRKY is an emerging player in the plant signaling regulation network. The interaction between the upstream regulator of the WRKY TF and the downstream target gene constitutes a complex regulatory network^25,26^.

Recently, several reports have shown that WRKY proteins have an obvious correlation with the regulation of anthocyanin biosynthesis. For example, *GbWRKY1* in *Gossypium barbadense* was proven to have a positive correlation with anthocyanin accumulation when expressed in *Arabidopsis thaliana*^27^. In a previous study, *AtWRKY75* responded to low phosphate (Pi) stress by decreasing anthocyanin accumulation in *A. thaliana* seedlings^28^. Moreover, the *Atwrky41* mutation resulted in increased anthocyanin content in *A. thaliana* rosette leaves^29^. *AtWRKY6* promotes PR1 promoter activity, which is related to senescence and pathogen defense, and the plant responds to abiotic and biotic stresses by decreasing anthocyanin accumlation^30^. Recently, it was reported that the WRKY TF *PhPH3* in petunia correlates with changes in the color of petals by playing a role downstream of the MBW complex^31^. Amato et al. also proved that *VvWRKY26*, a homologous gene of *PhPH3* in *Vitis vinifera*, induces the accumulation of flavonoids^23^. In apple, *MdWRKY40* is a key modulator in wounding-induced anthocyanin biosynthesis^32^. Yang et al. reported that the WRKY family was related to anthocyanin biosynthesis in red-skinned pear^33^. However, whether WRKYs are involved in anthocyanin biosynthesis by interacting with the TFs *PyMYB114* and *PybHLH3* in red pears is still unclear.

In this study, according to the transcriptome data of ‘Starkrimson’ and its green mutant pear at three fruit development stages, we screened two WRKY TFs and performed bioinformatics analysis. Furthermore, *PyWRKY26* or *PyWRKY31* with *PyMYB114* and *PybHLH3* were cotransformed into tobacco leaves and strawberry receptacles by a transient expression system. In addition, RT-qPCR analysis and dual-luciferase reporter system assays revealed the regulatory pattern of cotransformed *PyWRKY26* with its partners to activate the activity of anthocyanin biosynthesis- and transport-related structural genes. Furthermore, firefly luciferase complementation and Y2H assays confirmed the interaction of PyWRKY26 with PybHLH3. Our research reveals a potential mechanism of regulating anthocyanin biosynthesis in red-skinned pears, which will help elucidate the regulatory network to clarify anthocyanin accumulation in other species.

## Results

### Screening of the candidate WRKY genes by transcriptome data and bioinformatics analysis

**Fig. 1:**
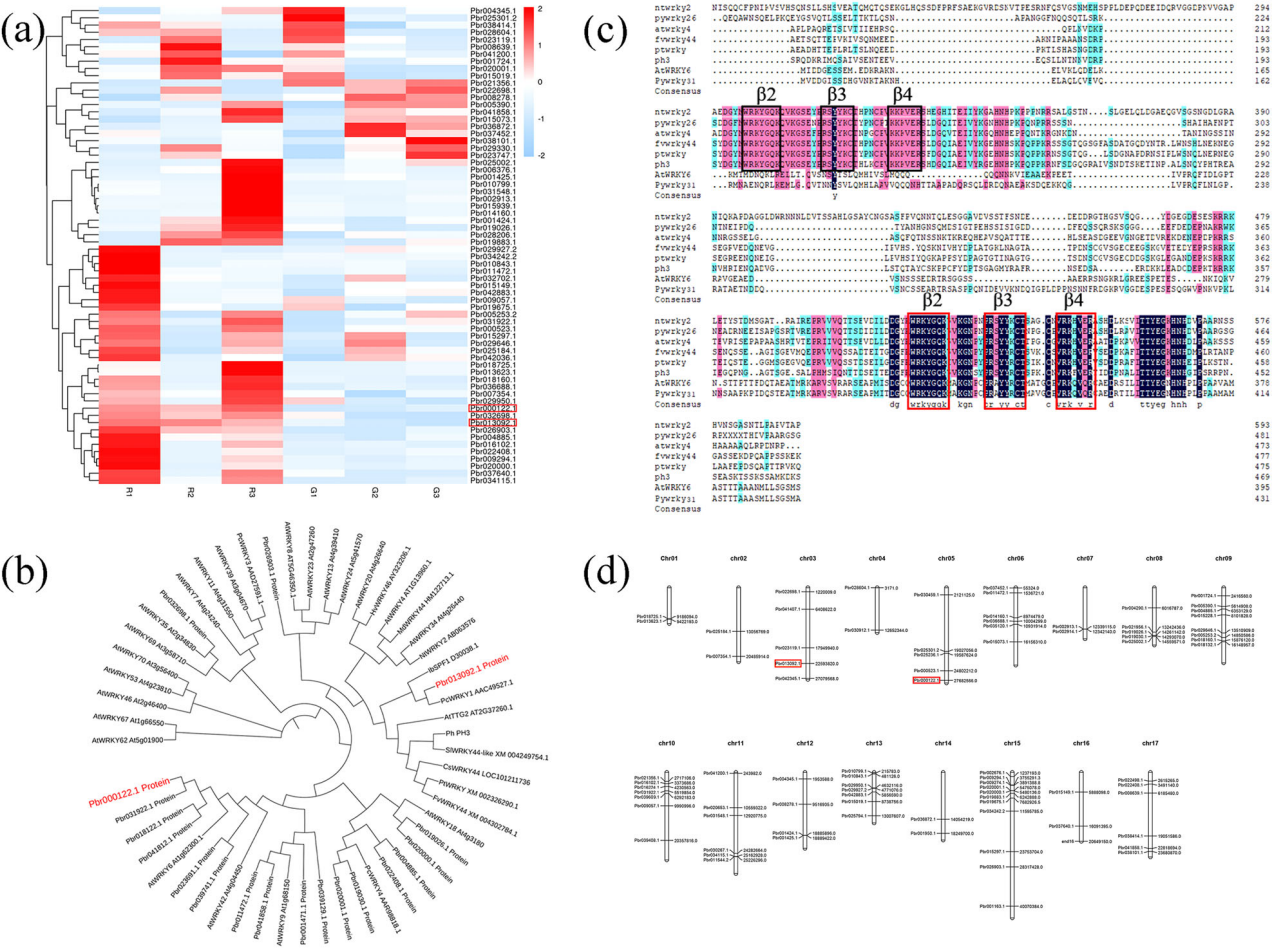
Screening and analyzing the candidate WRKY genes from transcriptome data of red-skinned pear ‘Starkrimson’ and its green mutant by bioinformatics. **a** WRKY gene transcript abundance in ‘Starkrimson’ (*Pyrus communis* L.) and its green mutant at different developmental stages. R1, R2, and R3 indicate the 40, 55, and 85 DAFB samples of the red-skinned pear of ‘Starkrimson’; G1, G2, and G3 indicate the 40, 55, and 85 DAFB samples of the green mutant of ‘Starkrimson’. **b** WRKY genes of different species are analyzed in the phylogenetic tree. The analysis of the alignments of the C-terminal WRKY domain was performed using the neighbor-joining method by the MEGA7 Program. **c** Multiple sequence alignment of *AtWRKY4* (At1g13960.1)*, PhPh3* (AMR43368)*, FvWRKY44* (XM_004302784.1)*, NtWRKY2* (AB063576)*, PtWRKY* (XM_002326290.1)*, PyWRKY26* (Pbr013092.1)*, PyWRKY31* (Pbr000122.1) and *AtWRKY6* (At3g58710). Identical, conserved and similar residues are shown in black, red and blue. WRKY-DNA domains are shown in squares with β2, β3, and β4. **d** Chromosomal localization of the WRKY genes in pear

A previous search by Yang et al. reported that the WRKY family was related to anthocyanin biosynthesis by RT-qPCR analysis in red-skinned pear^33^. To identify the function of WRKY genes in controlling anthocyanin biosynthesis in red pears, we screened 66 differentially expressed genes (DEGs) of the WRKYs and analyzed the transcriptome data of ‘Starkrimson’ pears and its green mutant at 40, 55, and 85 day after full bloom (DAFB). By analyzing the transcript abundance of 66 DEGs via a heat map, we found that Pbr000122.1, Pbr032698.1, Pbr013092.1, and Pbr026903.1 were more highly expressed in the three developmental stages of ‘Starkrimson’ fruits than in the green-skinned fruits (Fig. 1a). Furthermore, a phylogenetic tree was constructed using the neighbor-joining method and bootstrap analysis (1000 replicates) and MEGA7 software. The results indicated that the gene Pbr013092.1 (named *PyWRKY26*) has the most similar predicted protein sequences with homologous genes among all anthocyanin-related genes by a detailed phylogenetic analysis, which included the TFs *AtWRKY4*, *FvWRKY44*, *NtWRKY2*, *PhPH3*, *PtWRKY1*, and Pbr000122.1 (named *PyWRKY31*), which have high homology with *AtWRKY6* (Fig. 1b). In this phylogenetic analysis, other *PyWRKY* sequences are part of different clusters and groups, further indicating the gene function of *PyWRKY26* and *PyWRKY31*. Furthermore, analysis of the derived polypeptide alignment of *PyWRKY26*, *PyWRKY31* and other genes involved in anthocyanin biosynthesis, such as WRKY TFs, revealed the presence of WRKY motifs in all these genes (Fig. 1c).

Moreover, the members of the WRKY gene family were widely distributed on seventeen chromosomes in pear (Fig. 1d). As shown in Fig. 1d, most WRKYs were closely located to each other. This finding might indicate that tandem duplication events occurred in the WRKY gene family. The genes *PyWRKY26* (Pbr13092.1) and *PyWRKY31* (Pbr000122.1) located at Chr 3 and Chr 5 were explored in this study.

### Evaluating the correlation of *PyWRKY26/PyWRKY31* with anthocyanin accumulation and other factors regulating anthocyanin biosynthesis in pears

**Fig. 2:**
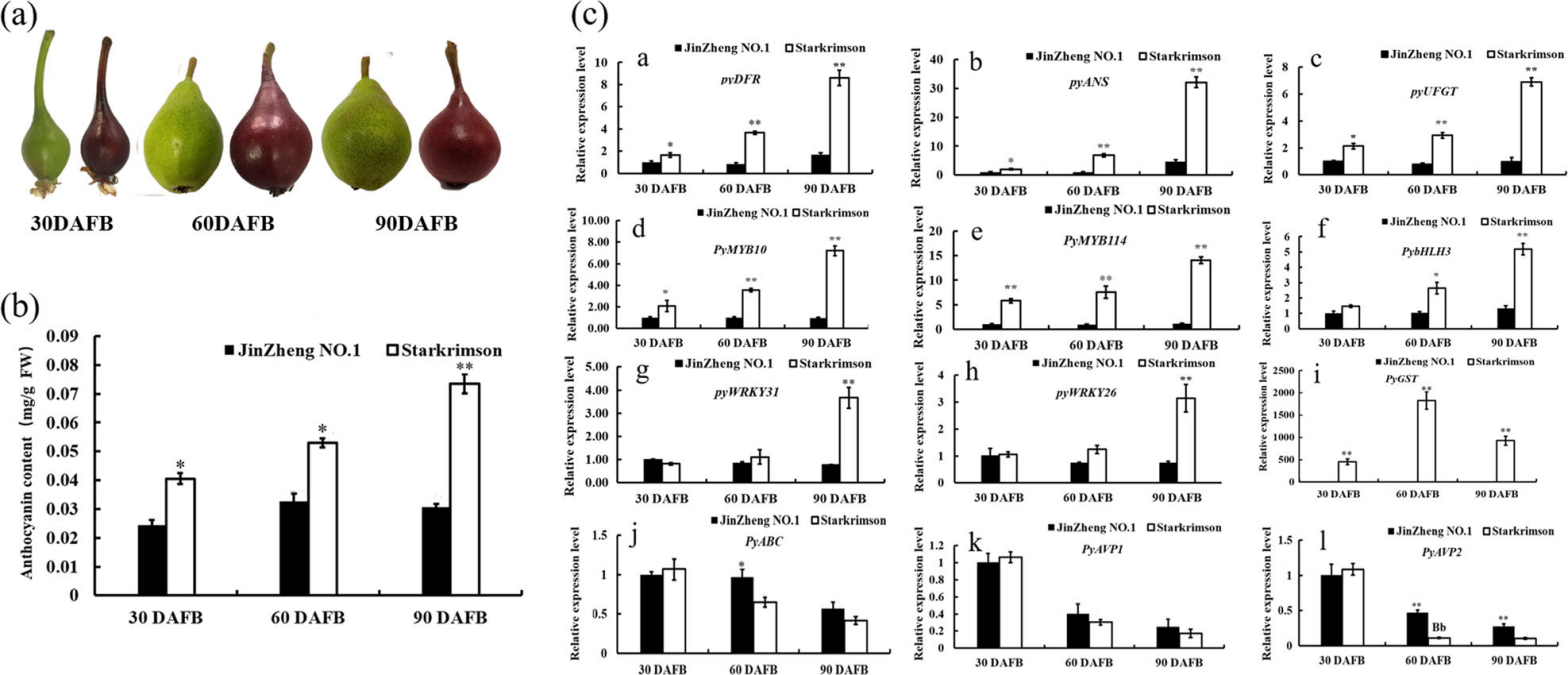
Evaluating the correlation of the candidate WRKY genes with anthocyanin biosynthesis and transport-related genes in ‘JinZheng No. 1’ and ‘Starkrimson’ pear cultivars. **a** The appearance of the ‘JinZheng No. 1’ and ‘Starkrimson’ pears at 30, 60, and 90 DAFB. **b** Determination of the anthocyanin contents in the ‘Starkrimson’ and ‘JinZheng No. 1’ pears at 30, 60, and 90 DAFB. **c** The expression level was analyzed by RT-qPCR for anthocyanin metabolic structural and regulatory genes. Significant differences are indicated at the level of *P* < 0.05 with lowercase letters. Highly significant differences are shown with uppercase letters (*P* < 0.01). Error bars are shown with the three biological replicates

To confirm the correlation of *PyWRKY26/PyWRKY31* with anthocyanin and anthocyanin-related TFs, we determined the anthocyanin contents in red-skinned ‘Starkrimson’ and green-skinned ‘Jinzheng No. 1’ pears at different developmental stages. The results showed that the anthocyanin contents increased with fruit development in the ‘Starkrimson’ pear and were higher than those of the ‘Jinzheng No. I’ pear (Fig. 2a). Furthermore, the expression levels of *PyDFR, PyANS, PyUFGT, PyMYB10, PyMYB114, PybHLH3, PyWRKY26, PyWRKY31*, and *PyGST* were largely higher in the ‘Starkrimson’ pear than in the ‘Jinzheng No. 1’ pear (except *PyABC transporter*, *PyAVP1* and *PyAVP2*) (Fig. 2b, a–h). There was an obvious positive correlation between the anthocyanin contents and the anthocyanin biosynthesis structural genes and TFs, such as *PyUFGT, PyGST, PyWRKY26, PyWRKY31*, *PyMYB114*, and *PybHLH3*, and a negative correlation between the anthocyanin contents and *PyABC transporter*, *PyAVP1* and *PyAVP2* (Fig. S1).

### Heterologous expression of *PyWRKY26/PyWRKY31* and other TFs induces anthocyanin accumulation in tobacco leaves

**Fig. 3:**
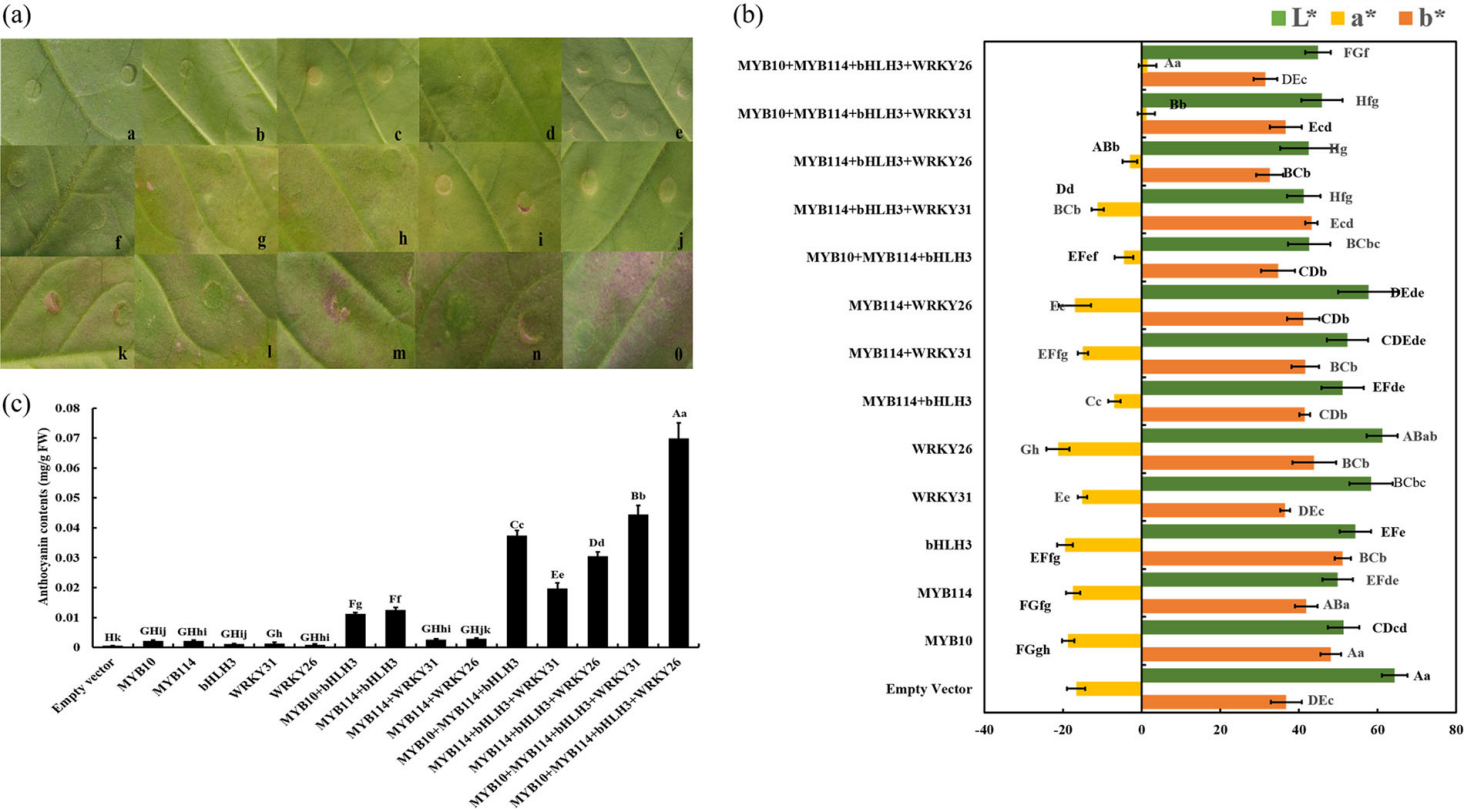
Cotransformation of *PyWRKY31/PyWRKY26* with its partners induced anthocyanin biosynthesis, as shown by transient expression assays in tobacco leaves. **a** The appearance of tobacco leaves 5 d after injection: a, *pSAK277*; b, *PyMYB10*; c, *PyMYB114*; d, *PybHLH3*; e, *PyWRKY31*; f, *PyWRKY26*; g, *PyMYB10* + *PybHLH3*; h, *PyMYB114* + *PybHLH3*; i, *PyMYB114* + *PyWRKY31*; j, *PyMYB114* + *PyWRKY26*; k, *PyMYB10* + *PyMYB114* + *PybHLH3*; l, *PyMYB114* + *PybHLH3* + *PyWRKY31*; m, *PyMYB114* + *PybHLH3* + *PyWRKY26*; n, *PyMYB10* + *PyMYB114* + *PybHLH3* + *PyWRKY31*; o, *PyMYB10* + *PyMYB114* + *PybHLH3* + *PyWRKY26*. **b** Differences in color are shown with the values of L*, a*, and b*. Significant differences are indicated at the level of *P* < 0.05 with lowercase letters. Highly significant differences are shown at the level of *P* < 0.01 with uppercase letters. Error bars are shown for six biological replicates. **c** Determination of the total anthocyanin contents in tobacco leaves with induced coloration

To verify the function of *PyWRKY31* and *PyWRKY26* in anthocyanin synthesis, we transiently transformed these genes into tobacco leaves. As shown in Fig. 3a, the empty vector pSAK277 was injected as a negative control, pigmentation was not observed when *PyWRKY31* or *PyWRKY26* alone was transformed with *PyMYB114*, and little pigmentation could be observed after cotransforming *PyMYB114* or *PyMYB10* with *PybHLH3*. The pigmentation was largely enhanced when *PyWRKY26* or *PyWRKY31* was cotransformed with *PyMYB114, PyMYB10*, and *PybHLH3*. Moreover, the anthocyanin contents in the tobacco leaves were analyzed by a colorimeter, and the changes in the L*, a*, and b* values were consistent with our expectations (Fig. 3b). When *PyWRKY26/PyWRKY31* was involved in cotransformation, the anthocyanin content in tobacco was significantly higher than that when only *PyMYB114, PyMYB10*, and *PybHLH3* were cotransformed (Fig. 3c) (*P* < 0.01). The above results indicated that cotransformation of *PyWRKY26/PyWRKY31* with *PyMYB10*, *PyMYB114*, and *PybHLH3* could significantly promote anthocyanin synthesis.

### Overexpression of *PyWRKY26/PyWRKY31* with other related TFs results in anthocyanin accumulation in strawberry receptacles

**Fig. 4:**
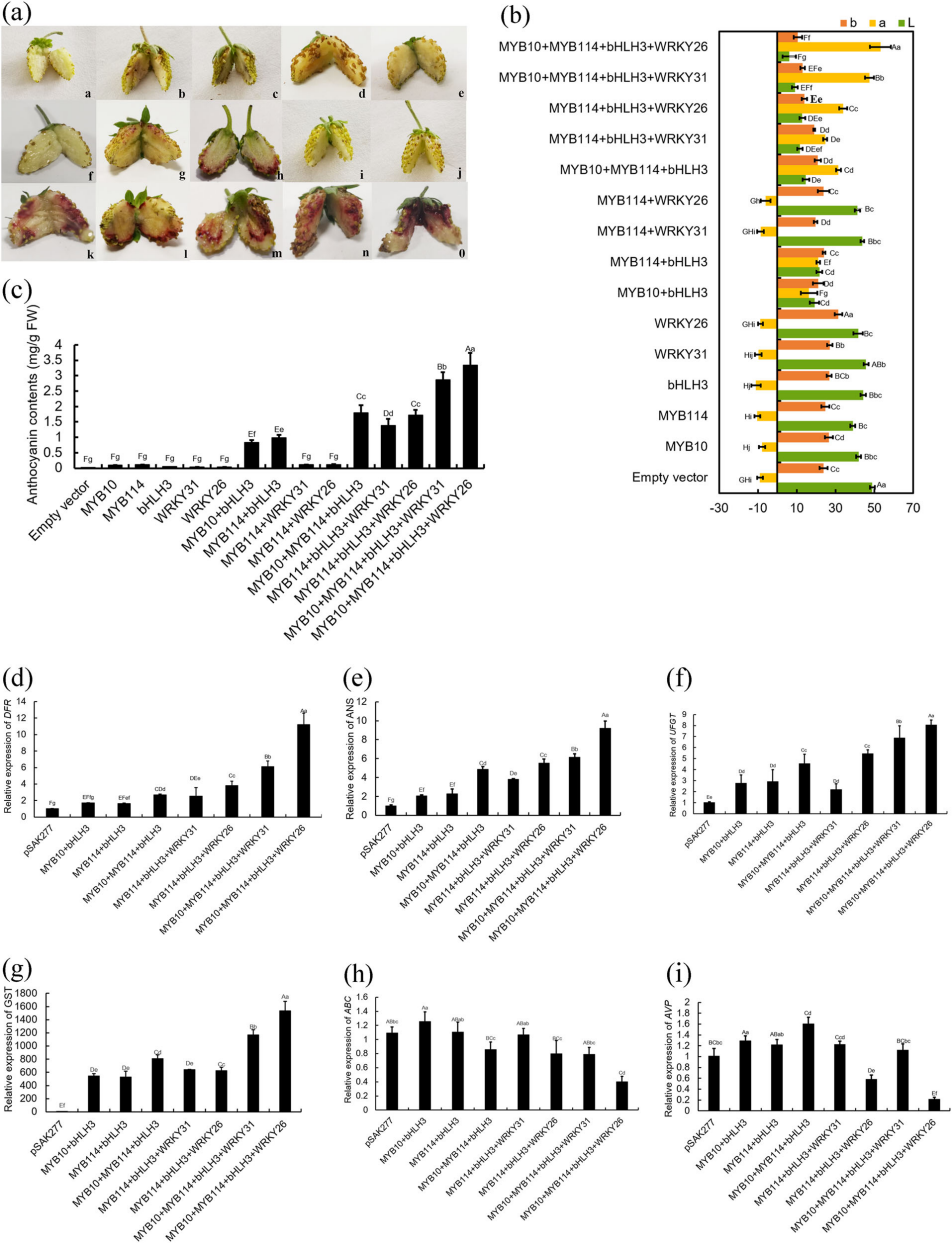
The functional analysis of *PyWRKY31/PyWRKY26* cotransformed with its partners, which resulted in anthocyanin biosynthesis, as shown by transient expression assays in strawberry receptacles. **a** The appearance of the strawberry receptacles 5 d after infiltration: a, *pSAK277*; b, *PyMYB10*; c, *PyMYB114*; d, *PybHLH3*; e, *PyWRKY31*; f, *PyWRKY26*; g, *PyMYB10* + *PybHLH3*; h, *PyMYB114* + *PybHLH3*; i, *PyMYB114* + *PyWRKY31*; j, *PyMYB114* + *PyWRKY26*; k, *PyMYB10* + *PyMYB114* + *PybHLH3*; l, *PyMYB114* + *PybHLH3* + *PyWRKY31*; m, *PyMYB114* + *PybHLH3* + *PyWRKY26*; n, *PyMYB10* + *PyMYB114* + *PybHLH3* + *PyWRKY31*; o, *PyMYB10* + *PyMYB114* + *PybHLH3* + *PyWRKY26*. **b** Differences in color are shown with the values of L*, a* and b*. Significant differences are indicated at the level of *P* < 0.05 with lowercase letters, and highly significant differences are shown at the level of *P* < 0.01 with uppercase letters. Error bars are based on six biological replicates. **c** Determination of the total anthocyanin contents. Error bars are based on three biological replicates. **d–i** RT-qPCR analysis of the expression levels of the genes *FvDFR*, *FvANS FvUFGT, FvGST, FvABC transporter* and *FvAVP*. Significant differences are indicated at the level of *P* < 0.05 with lowercase letters. Highly significant differences are indicated with uppercase letters at *P* < 0.01. Error bars show the SEs of the means (*n* = 3)

To further identify the function of *PyWRKY26* and *PyWRKY31* in anthocyanin synthesis, we performed transient transformation of strawberry receptacles. As shown in Fig. 4a, no pigmentation was observed in the empty vector pSAK277-transformed receptacles. The pattern of change was similar to that of tobacco leaves; *PyMYB114* + *PyWRKY31* or *PyMYB114* + *PyWRKY26* were cotransformed, and no pigmentation was observed. Some pigmentation could be observed when *PyMYB10* + *PybHLH3* or *PyMYB114* + *PybHLH3* were cotransformed. A deeper color change could be observed when *PyMYB114*, *PyMYB10*, and *PybHLH3* were coinjected. Meanwhile, the accumulation of anthocyanin was significantly higher when three TFs, *PyMYB114*, *PyMYB10*, and *PybHLH3*, were coinjected with *PyWRKY26/PyWRKY31*. In addition, the changes in the L*, a*, and b* values were influenced by the color change of the strawberry receptacles (Fig. 4b). When *PyWRKY26* was coinjected with *PyMYB114, PyMYB10*, and *PybHLH3*, the total anthocyanin content in strawberry was significantly higher than that with only the cotransformation of *PyMYB114, PyMYB10*, and *PybHLH3* (Fig. 4c) (*P* < 0.01). Meanwhile, when *PyWRKY31* was coinjected with *PyMYB114, PyMYB10*, and *PybHLH3*, the similar results for the anthocyanin contents were obtained. Overall, cotransformation of *PyWRKY26/PyWRKY31* with *PyMYB10, PyMYB114*, and *PybHLH3* can significantly enhance the anthocyanin biosynthesis in strawberry receptacles.

### RT-qPCR analysis of the expression levels of anthocyanin biosynthesis- and vacuolar transport-related genes in strawberry receptacles

To investigate the mechanism of *PyWRKY26/PyWRKY31* in regulating anthocyanin metabolism, we evaluated six structural genes of anthocyanin biosynthesis and transport in strawberry receptacles by RT-qPCR analysis. *FvDFR, FvANS*, and *FvUFGT* have been previously reported to control anthocyanin biosynthesis. The other three genes, *FvGST*, *FvABC transporter*, and *FvAVPs*, were identified as key genes involved in anthocyanin transport. As shown in Fig. 4d–f, cotransformation of *PyWRKY26/PyWRKY31* with *PyMYB10, PyMYB114*, and *PybHLH3* significantly enhanced the expression levels of the *FvDFR, FvANS, FvUFGT*, and *FvGST* genes. Meanwhile, *PyWRKY26* had a stronger upregulation than *PyWRKY31*. Compared with pSAK277 alone, the *FvAVP* and the *FvABC transporter* genes were significantly repressed when four TFs were cotransformed into the strawberry receptacles (Fig. 4h–i). RT-qPCR analysis suggested that cotransformation of four TFs, *PyMYB10, PyMYB114, PybHLH3*, and *PyWRKY26/PyWRKY31*, enhanced the anthocyanin synthesis and transport by upregulating the expression of the *FvDFR, FvANS*, *FvUFGT*, and *FvGST* genes and downregulating the expression of the *FvAVPs* and *FvABC transporter* genes in the strawberry receptacle.

### Validation of the interaction of the transcriptional regulatory complex by a dual-luciferase reporter system

**Fig. 5:**
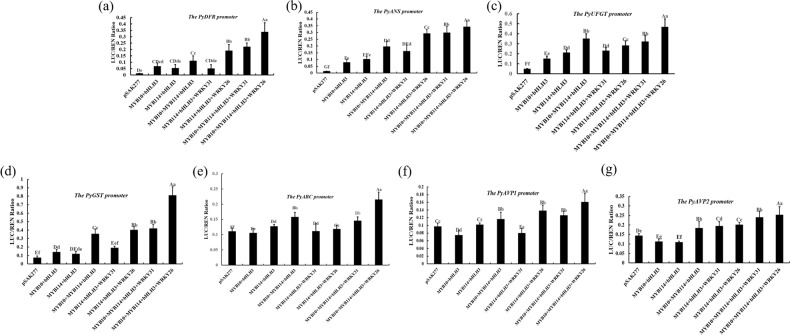
The transactivate activity of TFs *PyMYB10, PyMYB114, PybHLH3,* and *PyWRKYs* for anthocyanin biosynthesis and transport related genes was verified by a dual-luciferase reporter assay. The dual-luciferase reporter assay verified that *PyMYB10*, *PyMYB114*, *PybHLH3* and *PyWRKY26/PyWRKY3*1 cotransformation induces the activity of the *PyDFR* (**a**), *PyANS* (**b**), *PyUFGT* (**c**), *PyGST* (**d**), *PyABC transporter* (**e**) and *PyAVP1/2* (**f**, **g**) promoters. The LUC/REN value was used to express promoter activity. Error bars represent the SEs for three replicate reactions

A dual-luciferase reporter assay was used to verify the interaction of our candidate TFs with the structural genes, including *PyDFR, PyANS*, *PyUFGT, PyGST, PyABC transporter*, and *PyAVP1/2* in *Nicotiana tabacum*. The results showed that *PyMYB10* and *PyMYB114* cotransformed with *PybHLH3* could induce the activity of these promoters, and the additional TF *PyWRKY26/PyWRKY31* could significantly enhance the transactivation activity of the *PyDFR, PyANS*, *PyUFGT*, and *PyGST* promoters (Fig. 5a–d). Moreover, compared with that of pSAK277, the transactivation of the *PyABC transporter* and *PyAVP1/2* promoters was not obviously activated when *PyMYB114, PyMYB10, PybHLH3*, and *PyWRKY26/PyWRKY31* were cotransformed (Fig. 5e–g). These results indicated that *PyWRKY26/PyWRKY31* coregulates anthocyanin accumulation through coordination with the *PyMYB10* + *PyMYB114* + *PybHLH3* complex.

### Activation activity of *PyWRKY26* and *PybHLH3* on the *PyMYB114* promoter was verified

**Fig. 6:**
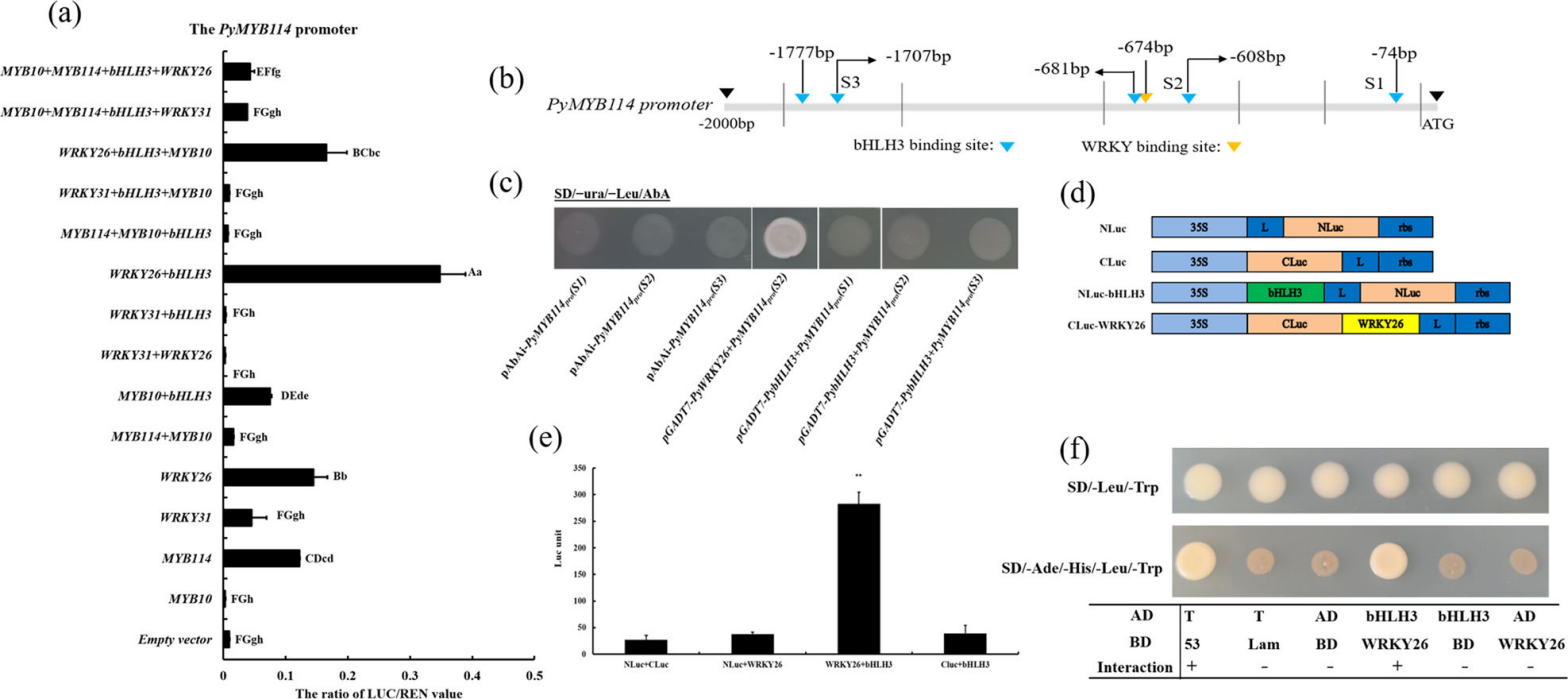
Verifying the regulatory pattern of TFs PyMYB114, PybHLH3, and PyWRKY26 in vivo. **a** The dual-luciferase reporter assay verifying *PybHLH3* and *PyWRKY26* cotransactivation activity. **b** A schematic of the *PyMYB114* promoter. The bar segments were analyzed by using the PLACE and PlantCare databases and are divided by solid lines (S1–S3). **c** Yeast one-hybrid assays between *PybHLH3/PyWRKY26* and the *PyMYB114* promoter. Positive colonies indicated strong specific interactions with the *PyMYB114* promoter segments. The colony name indicates the bait segment, and prey vectors were used. **d** Model of the NLuc, CLuc and NLuc/CLuc constructs. **e** Firefly luciferase complementation assays in young tobacco leaves. Error bars are shown with six biological replicates. **P < 0.01. **f** Verification of the interaction of PyWRKY26 and PybHLH3 in vivo

To further explore the relationship of the TFs *PyMYB114, PybHLH3*, and *PyWRKY26/PyWRKY31*, we cloned the upstream 2 kb promoter of *PyMYB114* and analyzed it by a dual-luciferase reporter assay in tobacco leaves. As shown in Fig. 6a, cotransformation with *PyWRKY26* and *PybHLH3* had a stronger activation effect on the promoter sequences of *PyMYB114* than other factors, including *PyWRKY31*. The binding of *PybHLH3* and *PyWRKY26* to the *PyMYB114* promoter was further verified by yeast one-hybrid technology. Promoter structure analysis revealed that multiple cis-regulatory elements were predicted by PlantCARE (Fig. 6b). The promoter segment baits were fused to the prey vectors pGADT7*-PybHLH3* and pGADT7*-PyWRKY26* and introduced into the Y1HGold yeast strain, and the results suggested that *PyWRKY26* could bind to the S2 fragments. However, we did not detect a direct association between *PybHLH3* and the promoter of *PyMYB114*, although several binding sites were located in different individual promoter regions (Fig. 6c).

### Verification of the interaction of PybHLH3 with PyWRKY26

For further identification of the interaction of *PybHLH3* with *PyWRKY26*, a tobacco-based firefly luciferase complementation assay was performed. *PybHLH3* was inserted into the N-terminal region of the firefly luciferase (NLuc), whereas *PyWRKY26* was linked to the C-terminal region of the firefly luciferase (CLuc) (Fig. 6d). Coexpression of the NLuc-*PybHLH3* and CLuc-*PyWRKY26* constructs showed the strongest capability to rescue intense luciferase enzyme activity (*P* < 0.01). Nevertheless, for the control constructs, including NLuc-*PybHLH3* with C and CLuc-*PyWRKY26* with Nluc, no obvious luciferase enzyme activity was observed (Fig. 6e).

Then, the interaction of PyWRKY26 with PybHLH3 was further verified by Y2H assays. The full-length coding sequences of *PyWRKY26* and *PybHLH3* were inserted separately into pGBKT7 and pGADT7. The results indicated that cotransformed *PyWRKY26* and *PybHLH3* in AH109 yeast cells resulted in healthy growth on the medium (Fig. 6f). Above all, the results suggested that *PyWRKY26* interacts with *PybHLH3* in vivo.

## Discussion

Anthocyanin, a common secondary metabolite in plants that exhibits antioxidant properties, gives plants a red or purple color. At present, an extensive body of literature has clearly demonstrated that the anthocyanin biosynthetic pathway is regulated at the transcriptional level by an MYB-bHLH-WD40 (MBW) regulatory complex in plants, and MYB in particular plays a vital role in anthocyanin synthesis. Compared with MYB, the bHLH TF has a significantly lower functional specificity, similar to *AtEGL3, AtGL3*, and *AtTT8*, and the three bHLH TFs have functional redundancy in the regulation of anthocyanin biosynthesis and trichome development^15–17^. In addition, WD40 plays an important role in enhancing the stability of the MBW complex, and the interaction between MYB and bHLH TFs is a prerequisite for specific recognition of DNA sequences^19,34^. In peach, the expression of *PpMYB10.1* was activated with partner TF helix-loop-helix proteins and *PpNAC1* and coregulated anthocyanin biosynthesis^35^. In pear, Yao et al. showed that *PyMYB114*/*PyMYB10*, which interacts with *PybHLH3* in tobacco leaves, strawberry receptacles and pears, could promote the biosynthesis of anthocyanins. Moreover, *PyERF3* promoted anthocyanin biosynthesis by coexpression with *PyMYB114* and *PybHLH3*^18^. In this study, we also demonstrated that *PyWRKY26*/*PyWRKY31* could be coexpressed with *PyMYB114* and *PybHLH3*, resulting in anthocyanin biosynthesis in tobacco leaves and strawberry receptacles (Figs 3, 4). Thus, the regulatory networks of color formation in red-skinned pears are highly complex, and whether and how the interaction of *PyWRKY26/PyWRKY31* with *PyERF3* regulates anthocyanin biosynthesis still need further study.

The important TF regulatory family WRKY plays important roles in various plant processes, such as plant growth and metabolism and senescence^35–37^. WRKYs were shown to exert their biological functions and regulate metabolite biosynthesis, especially that of secondary metabolites, through physical interactions with different proteins^38^. Verweij et al. reported that *PH3*, which encodes a WRKY protein of petunia, is a target gene of the AN11-AN1-PH4 complex, could bind to AN11 and is required together with the AN11-AN1-PH4 complex for the transcription of PH5 (which encodes an MYB protein), which regulates hair development, tannin accumulation, and mucilage production in *Arabidopsis*^31^. In our study, *PyWRKY26* largely enhanced anthocyanin accumulation after transient cotransformation with *PyMYB114* and *PybHLH3* compared with the two cofactors *PyMYB114* + *PybHLH3* (Figs. 3 and 4). This evidence proved that WRKY needs to interact with other partner factors to carry out its biological functions. The regulatory complex was consistent with Verweij et al.^31^, although *PyWRKY26* could regulate anthocyanin accumulation, while PH3 regulates tannin accumulation and mucilage production. Thus, TF WRKYs are involved in the formation of different secondary metabolites. In addition, we further explored the target genes of *PyWRKY26* and *PyWRKY31*, and cotransformation with *PyWRKY26* and *PybHLH3* had a stronger activation effect on the promoter sequences of *PyMYB114* than other factors. *PyWRKY26* directly activated the promoter sequences of *PyMYB114* (Fig. 6b, c), but *PyWRKY31* could not bind to the *PyMYB114* promoter (Fig. 6a). Further, the interaction of PyWRKY26 with PybHLH3 was proven by firefly luciferase complementation and Y2H assays (Fig. 6d–f). Therefore, *PyWRKY26* and *PyWRKY31* could regulate color formation, but the downstream target genes were different.

Generally, TFs forming regulatory complexes regulate anthocyanin accumulation by a series of anthocyanin biosynthesis- and transport-related structural genes. In this study, the expression levels of *PyDFR, PyANS, PyUFGT*, and *PyGST* in red-skinned pear were generally higher than those in green-skinned pear, and the expression patterns of *ABC transporters* and *AVPs* were opposite (Fig. 2). Furthermore, cotransformation of *PyWRKY26* with its partners in strawberry receptacles showed that the expression of *FvDFR, FvANS, FvUFGT*, and *FvGST* was upregulated and *FvABC transporter* and *FvAVPs* were downregulated (Fig. 4). The dual-luciferase reporting system also demonstrated a similar conclusion (Fig. 5). It has been widely reported that DFR, ANS, and UFGT catalyze anthocyanin biosynthesis. GST has been shown to be involved in anthocyanin transport in strawberry^39^. Recently, the ATP-binding cassette transporter *AtABCC2* was reported to be involved in the vacuolar transport of anthocyanins and other flavonoids in *Arabidopsis*^40^. In a previous study, GST acted as a carrier of anthocyanins and mobilized anthocyanins from the cytoplasm to the tonoplast by the ABC transmembrane transporter^8,9^. Many secondary transporters and channels, such as malate transporter, MATE-type transporters and ABC transporters, exist on the tonoplast, and the activities of these transporters are directly or indirectly dependent on the proton gradient generated by V-ATPase and V-Ppase^10,11,41–43^. The H^+^-pumping activities of vacuolar H^+^-ATPase (VHA) and pyrophosphatase (VHP) have an extremely important role in the transport of anthocyanins, malate and other metabolites^44^. These reports were consistent with the findings of our study showing involvement in the vacuolar transport of anthocyanins, but previous reports did not present evidence of the relationship between transport-related genes and the transcription regulatory complex. Moreover, we confirmed that the regulatory mode of the *PyABC transporter* and *PyAVPs* is different from that of *PyGST*, and we speculate that there is a difference in the timing between the anthocyanin synthetic process and transport process in plant cells. When the synthetic process was significantly activated, which results in anthocyanin accumulation, *PyGST* participates in transport over time, but the *PyAVP* and *PyABC transporter* genes are not activated. Anthocyanin biosynthesis and anthocyanin transport may be independent processes, although they are regulated by the same transcription complex; furthermore, they are not expressed simultaneously. The molecular mechanism by which transporter genes participate in anthocyanin transport still requires further research.

## Conclusion

In this study, the TFs *PyWRKY31* and *PyWRKY26* with their partners *PyMYB10, PyMYB114*, and *PybHLH3* were cotransformed into tobacco leaves and strawberry receptacles and resulted in increased anthocyanin contents. Furthermore, we confirmed that this cotransformation activated the activity of *PyDFR*, *PyANS*, and *PyUFGT* in anthocyanin biosynthesis and *PyGST* in anthocyanin transport instead of the *PyABC* transporter and *PyAVPs*. Moreover, firefly luciferase reporter assays and yeast expression assays showed that the interaction of *PyWRKY26* and *PybHLH3* could cotarget the *PyMYB114* promoter and that *PyWRKY26* directly activates the promoter sequences of *PyMYB114*, which resulted in anthocyanin accumulation in red-skinned pear. This research provides novel insight into the regulatory network of anthocyanin accumulation and contributes to improving the appearance quality of red-skinned pears.

## Materials and methods

### Plant materials

The green-skinned pear ‘JinZheng No. 1’ and red-skinned pear ‘Starkrimson’ used in this study were collected from the orchard of the Institute of Pomology in Shandong Province during the 2018 growing season. Tobacco (*N. tabacum*) and diploid strawberry (*Fragaria vesca*, called ‘Yellow Wonder’ 5AF7) were used in this study for the transient transformation experiments, dual-luciferase reporter system assays and firefly luciferase complementation assays, and they were cultivated in an intelligent incubator with a 16 h photoperiod and a 21 °C/17 °C day/night temperature. Young tobacco leaves and strawberry receptacles ~2 weeks after flowering were infiltrated for the experiment, and observations at 4–6 d after injection were performed. The tobacco leaves and strawberry receptacles were collected and chopped, frozen with liquid nitrogen and stored at −80 °C.

### Extraction and determination of the anthocyanins in tobacco leaves and strawberry

Anthocyanins were extracted according to the method of Yang et al.^33^. Approximately 0.2 g of the fruit skin of ‘JinZheng No. 1’ and ‘Starkrimson’, tobacco leaves or strawberry receptacles stored at −80 °C were ground to powder in liquid nitrogen and then homogenized with 1 ml of cold methanol containing 0.1% HCl at 4 °C for 24 h, and the homogenate was allowed to incubate in the dark at 4 °C for 24 h. Then, the supernatant was collected by centrifugation at 12,000 rpm for 20 min. The absorbance of the anthocyanins was measured at wavelengths of 530, 620, and 650 nm using a Multiskan Spectrum (Thermo Scientific Multiskan GO 1510, Finland). There were three biological replicates per sample, and the total content of anthocyanin per sample fresh weight was calculated according to the following formula: OD = (A_530_ − A_620_) − 0.1 × (A_650_ − A_620_).

### Total RNA extraction and gene transcript abundance analysis by RT-qPCR

Fruit skin of ‘JinZheng No. 1’ and ‘Starkrimson’ and strawberry receptacles were ground into fine powder in liquid nitrogen. Total RNA of the powder samples was extracted in an ice bath. First strand cDNA synthesis was performed using Prime Script RT Master Mix (DRR036A, TaKaRa, China). RT-qPCR was conducted using SYBR^®^ Premix Ex Taq^TM^ II (DRR081A, TaKaRa, China) in a 10 μl volume. The amplification program was as follows: one cycle of 30 s at 95 °C, followed by 40 cycles of 15 s at 95 °C and 30 s at 60 °C; a strawberry housekeeping gene (gene11892) was used as an internal control. The relative expression level of the genes was calculated using the 2^(-ΔΔCt)^ method. All analyses and error bars were determined using three biological replicates. The primer sequences for RT-qPCR are listed in Table S1.

### Genes cloning and overexpression vector construction

The gene sequences of *PyWRKY26* and *PyWRKY31* cloned from the cDNA of the red-skinned pear cultivar ‘Starkrimson’ were used in the following experiment. PCR amplification was conducted using TransStart FastPfu DNA Polymerase (AP221-01, Transgen, China) and the primer sequences listed in Table S2. The *PyWRKY26* and *PyWRKY31* genes were inserted into the pSAK277 vector under the control of the 35 S promoter with *EcoR*I and *Xho*I^45^. The integrated constructs were transformed into the *Agrobacterium tumefaciens* strain GV3101 using the chemical method, and the cells were incubated at 28 °C for 2 d. A description of the infecting is in Yao et al.^18^. The specific method of infiltration experiments was described by Voinnet et al.^46^. Tobacco leaves and strawberry receptacles were collected for anthocyanin measurement and RNA extraction at 5–7 d after infiltration. Empty vector infiltrations (pSAK277) were used as negative controls.

### Dual-luciferase reporter system assays

For the dual-luciferase reporter assay, the upstream promoter sequences of *PyMYB114* (2.0 kb), *PyAVP1/2* (1.8 kb), *PyDFR* (2.0 kb), *PyANS* (2.0 kb), *PyUFGT* (1.8 kb), *PyABC* transporter (2.0 kb), and *PyGST* (2.0 kb) were cloned and inserted into the pGreen II 0800-LUC vector with the primers listed in Table S2. The recombinant plasmids were transformed into the *Agrobacterium* strain GV3101 (PM90) with the pSoup helper plasmid. The TFs *PyWRKY26* and *PyWRKY31*, *PyMYB114*, *PyMYB10*, and *PybHLH3* were mixed with the promoter sequences (the ratio 1:9, v/v) and then injected into young tobacco leaves for transient cotransformation expression analysis^18^. According to the manufacturer’s instructions, the ratio of transactivation activities of firefly luciferase and renilla luciferase was tested by the Dual-Luciferase^®^ Reporter Assay System (E1910, Promega, USA).

### Firefly luciferase complementation assay

Firefly luciferase complementation assays were performed according to the method of Chen et al.^47^. Gene sequences of *PybHLH3* (with no stop codon) were amplified and linked with pCAMBIA1300-NLuc, and the coding sequences of *PyWRKY26* were cloned and linked with the pCAMBIA1300-CLuc vector. The primer sequences are listed in Table S2. Then, the cells were transformed into *Agrobacterium* GV3101 and cultured using a selection medium containing kanamycin. The cells were grown to OD_600_ 0.6, and then, bHLH3-NLuc and *PyWRKY26*-CLuc were mixed 1:1 by volume and infiltrated into the tobacco leaves. Leaf disks (exactly 2 cm in diameter) were punched adjacent to the infiltration site, and the firefly luciferase activity was determined by a Steady-Glo^®^ Luciferase Assay System (E2510, Promega, USA).

### Yeast one-hybrid assay

To identify transcriptional regulators of *PyMYB114* by yeast one-hybrid assays, we used the ~300 bp promoter segments, corresponding to the S1–S3 sequences. The promoter fragments were inserted into the pAbAi vector, and the *PybHLH3* and *PyWRKY26* genes were cloned into the pGADT7 vector. In a preliminary filter, self-activation of the bait vectors was tested on SD/-ura+AbA^100^, SD/-ura+AbA^200^, and SD/-ura+AbA^400^ plates; the prey vectors pGADT7*-PybHLH3* and pGADT7*-PyWRKY26* were tested on SD/-Leu plates. The promoter segment baits were fused to the prey vectors pGADT7*-PybHLH3* and pGADT7*-PyWRKY26* and introduced into the Y1HGold yeast strain and tested on SD/-ura+AbA plates at 30 °C for 3 d. The primer sequences used for vector construction are listed in Supporting Information Table S1.

### Yeast two-hybrid assay

According to the Matchmaker^®^ Gold Yeast Two-Hybrid System (Clontech, HTTP:// www.clontech.com/), a Y2H assay was performed to test for protein interactions. The *PyWRKY26* and *PybHLH3* genes were inserted separately into pGBKT7 and pGADT7 and then cotransformed into the yeast strain AH109. The transformants were selected on SD/-Leu/-Trp medium and tested on SD/-Leu/-Trp/-His/-Ade medium. Meanwhile, pGADT7-T and pGBKT7-Lam or pGADT7-T and pGBKT7-53 were cotransformed as negative and positive controls.

### Statistical analysis

All samples were assessed at least three times independently, and all data are represented as the mean ± SD. Statistical analysis was performed by Student’s *t*-test and one-way ANOVA. Significance was indicated by asterisks * (*P* < 0.05) or ** (*P* < 0.01) or different letters.

## Supplementary information

Table S3

Figure S1

Figure S2

Table S1

Table S2
