# Supplementary figures and images for: PyWRKY26 and PybHLH3 cotargeted the PyMYB114 promoter to regulate anthocyanin biosynthesis and transport in red-skinned pears
Source: Hortic Res. 2020 Mar 15;7:37. doi: 10.1038/s41438-020-0254-z (PMC7072072; doi:10.1038/s41438-020-0254-z)

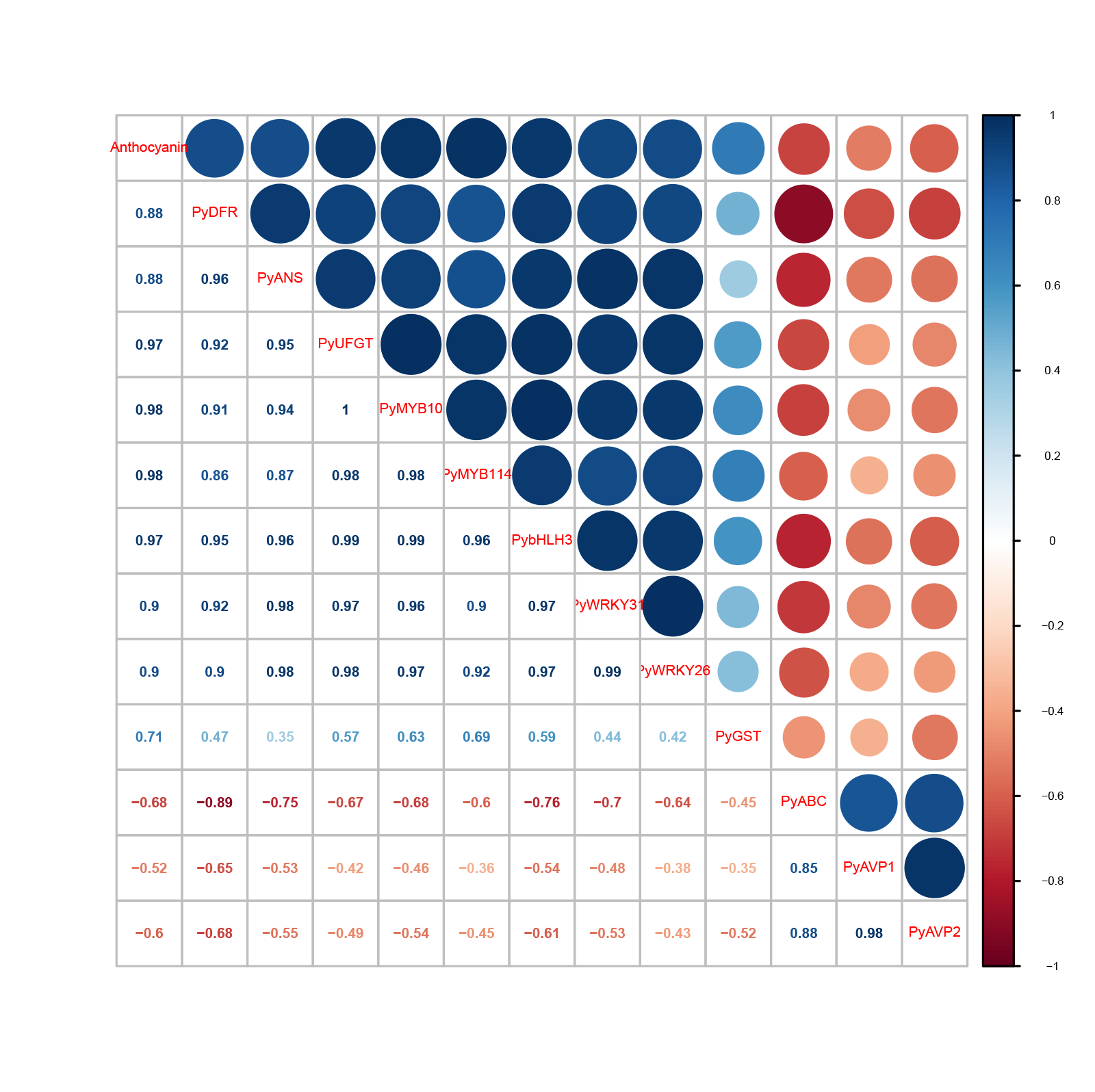

Supplement: Supplementary file 2 — Figure S1 [file 41438_2020_254_MOESM2_ESM.png]

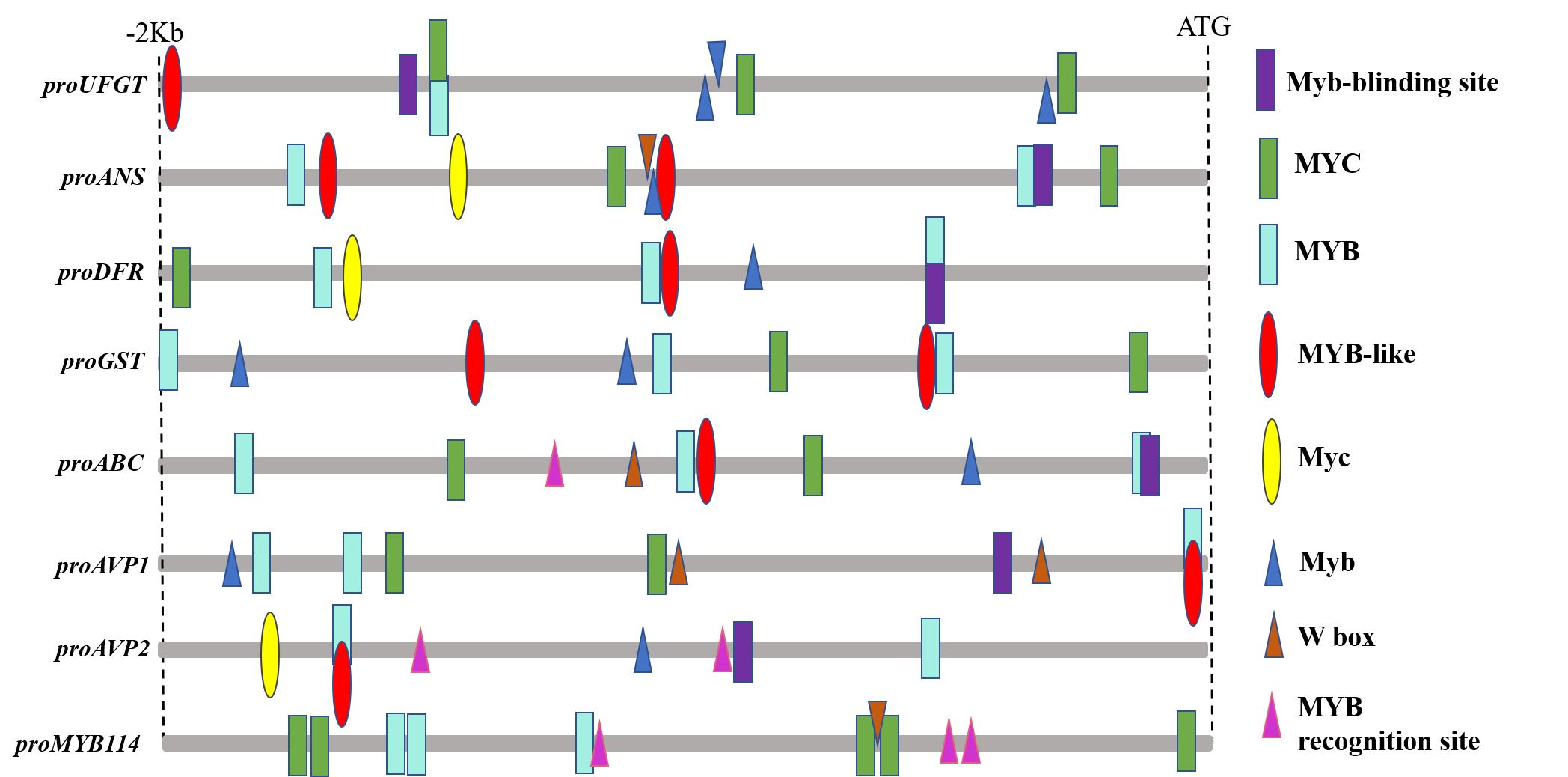

Supplement: Supplementary file 3 — Figure S2 [file 41438_2020_254_MOESM3_ESM.png]
