# Supplementary material for: PyWRKY26 and PybHLH3 cotargeted the PyMYB114 promoter to regulate anthocyanin biosynthesis and transport in red-skinned pears
Source: Hortic Res. 2020 Mar 15;7:37. doi: 10.1038/s41438-020-0254-z (PMC7072072; doi:10.1038/s41438-020-0254-z)
Supplement: Supplementary file 4 — Table S1 [file 41438_2020_254_MOESM4_ESM.docx]

**Table S1** The list of primers used in this study for qRT-PCR.

| **Primer name** | **Primer sequence (5'-3')** |
| --- | --- |
| FvDFR-F | CACGATTCACGACATTGCGAAATT |
| FvDFR-R | GAACTCAAACCCCATCTCTTTCAGCTT |
| FvANS-F | GAAGTGCGTACCCAACTCCATCGT |
| FvANS-R | ACCTTCTCCTTGTTGACGAGCCC |
| FvUFGT-F | TTTGGTTCGGTGCTCATA |
| FvUFGT-R | AGCATCTGTCCCATCTGGT |
| FvGST-F | CAAGTTCCAGCAATCGAAGA |
| FvGST-R | TGGGAAGGATCACAAGTTGA |
| FvABC-F | GAGTACTGTGATTGCGTTGATC |
| FvABC-R | GACTTCAAGTTCAAACGTCGAA |
| FvAVP-F | TGCTTCAATTTCCTCATTTGGG |
| FvAVP-R | GAAATCCATGTAACGATCGCAA |
| Housekeeping-F | AGCCTAACGCAGAGGTTCCAAA |
| Housekeeping-R | GCAGCCCACATTGAAGGGTCTATAGT |
| PyMYB114-F | GCCACATCCGTCATAAGACCTC |
| PyMYB114-R | GCCACTCATGTGTAACCCTTC |
| PyMYB10-F | GACCAATGTGATAAGACCTCAGCC |
| PyMYB10-R | CCGTTCTTTGTTGACGACGAC |
| PybHLH3-F | TTGTGGAGGGAAGTGGCGGT |
| PybHLH3-R | AGCTCCCTAAGTGTTTGCATCAC |
| PyDFR-F | GGTTTCATCGGCTCTTGGC |
| PyDFR-R | CCTTCTTCTGATTCGTGGGGT |
| PyUFGT-F | CCTTCCCTTTTGCCACTCA |
| PyUFGT-R | CAGCCACATCGTACACCCTTA |
| PyANS-F | GAGCAGAAGGAGAAGTAT |
| PyANS-R | ACAGTGGAAGAAGTAGTC |
| PyWRKY26-F | GCTTCTTGACTCTCCTGTTCTC |
| PyWRKY26-R | GTGGTTCTTGCTCTCCTGTT |
| PyWRKY31-F | GACCTCACCACATCTCCATTAC |
| PyWRKY31-R | GGGTTGCCCTGGGAAATTA |
| PyGST-F | TGGGCAAGTTCCAGTTGTAG |
| PyGST-R | CCTCCAGAGTTGTTCCCAATAG |
| PyABC-F | CCTTCGAGGCCAGATGTTATT |
| PyABC-R | AACAGTGCTCTTCCCTGAAC |
| PyAVP1-F | CTCTTGTGTCCCTTGCTCTATT |
| PyAVP1-R | CGCCCACTAACAAACCAATAATC |
| PyAVP2-F | CTCGTTCCTCTTCACGGAATAC |
| PyAVP2-R | ATGCACGGCTACTTGTACTAAA |
| Actin-F | GACCACCACTGGTCATCTTATC |
| Actin-R | CATCTCAGCAGCTTCCTTCTC |
